# Construction of a quadruple gene-deleted vaccine confers complete protective immunity against emerging PRV variant challenge in piglets

**DOI:** 10.1186/s12985-022-01748-8

**Published:** 2022-01-25

**Authors:** Leqiang Sun, Yajie Tang, Keji Yan, Huawei Zhang

**Affiliations:** 1 State Key Laboratory of Agricultural Microbiology, Huazhong Agricultural University, Wuhan, 430070 Hubei China; 2 Henan Joint International Research Laboratory of Veterinary Biologics Research and Application, Anyang Institute of Technology, Anyang, Henan China

**Keywords:** Pseudorabies virus, CRISPR/Cas9 technology, gI/gE/US9/US2, Safety and efficacy

## Abstract

**Background:**

Pseudorabies virus (PRV) causes Aujeszky’s disease or pseudorabies (PR) in pigs worldwide, which leads to heavy economic losses to the swine industry. Pigs are the natural host, meanwhile, animals such as dogs, cats, foxes, rabbits, cattle and sheep are susceptible to infection. In 2011, the emerging PRV variant led to the outbreak of PR in Bartha-K61 vaccinated pigs. The PR outbreaks demonstrated that the Bartha-K61 vaccine did not provide full protection against the emerging PRV variant. It is widely believed that PRV live attenuated vaccine could control PRV infection.

**Methods:**

In this study, we developed a novel PRV live attenuated vaccine by deleting its gI, gE, US9, and US2 genes through CRISPR/Cas9, which was named PRV GDFS-delgI/gE/US9/US2.

**Results:**

Safety experiments confirmed that PRV GDFS-delgI/gE/US9/US2 was safe for 5- to 7-day-old suckling piglets. Piglets immunized with the PRV GDFS-delgI/gE/US9/US2 vaccine did not produce PRV gE-specific antibodies but could generate PRV gB-specific antibodies and high neutralizing titers against the PRV GDFS strain (variant PRV strain) or PRV Ea strain (older PRV strain). After challenge with the emerging PRV GDFS variant, none of the piglets immunized with the PRV GDFS-delgI/gE/US9/US2 vaccine showed any clinical signs, and their rectal temperatures were normal. Moreover, the autopsy and histopathological analyses revealed that the piglets in the PRV GDFS-delgI/gE/US9/US2 vaccine group did not show apparent gross or pathological lesions. Furthermore, the piglets in the PRV GDFS-delgI/gE/US9/US2 vaccine groups did not present weight loss. According to the criteria of the OIE terrestrial manual, the results of the experiment confirmed that the PRV GDFS-delgI/gE/US9/US2 vaccine could provide full protection against the emerging PRV variant strain in piglets.

**Conclusions:**

The PRV GDFS-delgI/gE/US9/US2 strain is a potential new live attenuated vaccine against emerging PRV variant strain infections in China.

## Introduction

Pseudorabies virus (PRV) is a member of the family Herpesviridae, subfamily Alphaherpesvirinae, and genus *Varicellovirus* [1]. It can infect many domestic and wild animals (such as pigs, dogs, cats, foxes, rabbits, cattle and sheep). Pigs are its natural host and reservoir, and the main source of infection. PRV infection causes high mortality in newborn piglets, respiratory symptoms and growth retardation in finishing pigs, and reproductive failure in sows, which lead to heavy economic losses in the swine industry [1–3].

The Bartha-K61 attenuated vaccine is considered safe and effective and plays an important role in protection against SuHV-1 infection [4]. Bartha-K61 was developed by in vitro continuous-passage culture, and the sequencing of the Bartha-K61 genome shows that almost 3500 bp of a large fragment in the genome is deleted, including the complete gE and US9 genes and parts of the gI and US2 genes [4, 5]. The Bartha-K61 vaccine has been widely used to control PR in North America and some European countries in the past few decades [6]. gI, gE, US9, and US2 are genes not essential for PRV replication and not the protective antigen of PRV. gE and gI genes are both important virulence factors and anterograde spread within the nervous system. US9 is a type II tail-anchored membrane protein while glycoproteins E and I (gE and gI) are type I membrane proteins that function in the context of a gE/gI heterodimer. US2 is a tegument protein that function in membrane associated protein [7].

The Bartha-K61 vaccine was imported from Hungary to China in 1979. It is widely used in China and played a critical role in the control of PR from 1990 to 2010 [1]. However, since 2011, outbreaks of infection with the variant PRV have been confirmed in most regions of China [3, 8–12]. Since the PRV epidemic, many previously PRV-negative pig farms have become positive, causing significant economic losses in the pig industry. Since the outbreak of the variant PRV among Bartha-K61 vaccinated pigs in large-scale pig farms, several studies have shown that the Bartha-K61 vaccine cannot provide full protection against the emerging PRV variants [3, 9]. Several studies have demonstrated that genetic mutations can be observed in the genome of the variant PRV [13–16]. Phylogenetic analysis indicates that the new PRV isolates belong to genotype II and are different from the classical PRV strains, such as NIA3, Becker, Bartha, and Kaplan [2].

Gene-deleted inactivated vaccines and live attenuated vaccines based on emerging PRV variants have been developed in China. For instance, JS-2012-△gI/gE, rPRVTJdelgE, rPRVXJ-delgI/gE-EGFP, PRV-HNX TK-/gE, rPRVTJ-delgE/gI/TK, vPRVHN1201TK-/gE-/gI-, rSMX△gI/gE△TK, and rZJ01△TK/gE/gI have been con-structed [17–25]. However, the levels of immune protection provided indicate that different doses of PRV vaccines or PRV challenge and different PRV strains can lead to different effects. In general, the live-attenuated PRV vaccines are demonstrably more efficacious than the inactivated PRV vaccines.

In our laboratory, three variant PRV strains were isolated from aborted fetus samples from three pig farms of Bartha-K61 vaccinated pigs. These variant PRV strains could cause 80%–100% mortality in 50–60 day-old piglets. In this study, we constructed a novel gI/gE/US9/US2-deleted attenuated vaccine strain on the basis of the variant PRV strain (PRV GDFS), referring to the deletion of the PRV Bartha-K61 strain using CRISPR/Cas9 technology. The safety and effectiveness of the PRV GDFS-delgI/gE/US9/US2 vaccine were investigated in a suckling piglet model. It was found that PRV GDFS-delgI/gE/US9/US2 had no pathogenicity for suckling piglets and conferred complete protection against PRV infection in suckling piglets. Thus, PRV GDFS-delgI/gE/US9/US2 is a potential new attenuated vaccine strain against emerging PRV variant infection.

## Materials and methods

### Virus and cells

PRV-GDFS (GenBank No. MH521043) was isolated from Guangdong Province of China in 2019, which had belonged to the variant PRV strain. PK-15 cells (ATCC No. CCL-33) were cultured in Dulbecco’s modified essential medium (DMEM; Invitrogen, USA) with 10% fetal bovine serum (HyClone, USA) and 5% CO2 at 37 °C in a humidified incubator.

### Construction of transfer plasmid and sgRNA plasmids

**Fig. 1:**
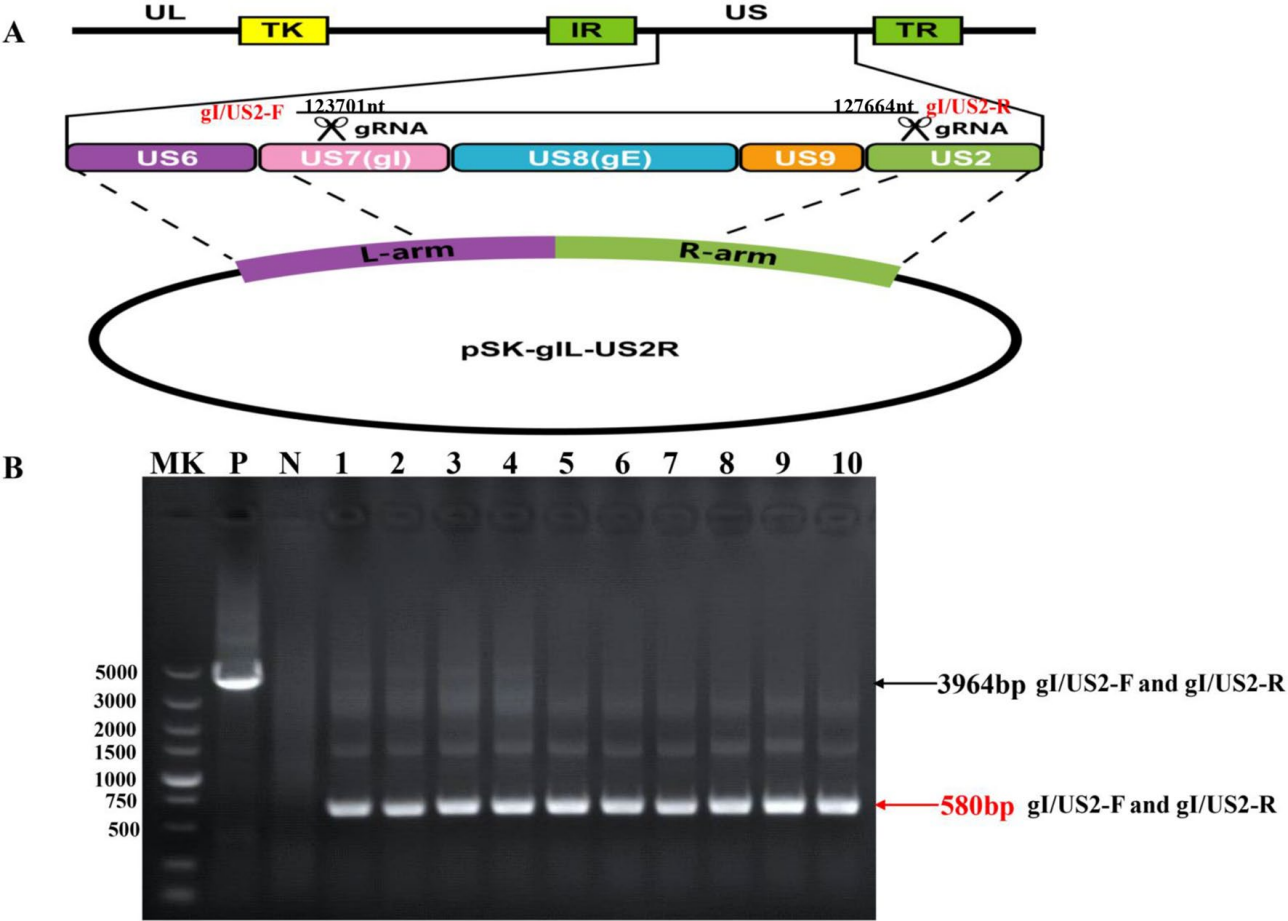
Schematic diagrams of the PRV GDFS-delgI/gE/US9/US2 strain. **A** Diagram of the PRV gI/gE/US9/US2 gene deletion. The transfer plasmid pSK-gIL-US2R was constructed for homologous recombination with co-transfection of two sgRNAs and PRV GDFS genome. **B** Identification of the plaque-purified viruses by PCR with specific primers (gIF and US2R). PRV GDFS was used as the positive control (P), and DMEM was used as the negative control (N)

A transfer plasmid was constructed by using two segments flanking the gI and US2 genes (Fig. 1A). The fragments of gI-L and US2-R were amplified by polymerase chain reaction (PCR) with gD-F/gI-R and US2-F/US2-R primers. Then, the two PCR products were inserted into the pBluescript II SK (pSK) vector. Finally, the transfer plasmid pSK-gIL-US2R was obtained, as the recombination homologous arms. The pCas9-gI targeting site was 5′-TACGACCCCGCGTCCCCCG-3′, and the pCas9-US2 targeting site was 5′-GGGGTGACGGCCATCACCG-3′. The guide RNAs were synthe-sized and cloned into the PX335 plasmids [26]. All the sequences of the primers and sgRNAs are listed in Table 1.

**Table 1:** Sequences of oligonucleotides used in this study

Primer	Sequence	Details
gD-F	TCTCGAGGAGGACCCGTGCGGGGTGGTGGCG	Left Homologous arm
gI-R	TGAATTCGCAGGCGCGCTTGGGGTCGAGGCGC	
US2-F	TACTAGTGCTGGACACGGAGTGGTCGTCCGTCC	Right Homologous arm
US2-R	TGCGGCCGCGTGAGGCGGGCCGCGCCCCGCTCT	
sgRNA-gI-F	CACCGTACGACCCCGCGTCCCCCG	sgRNA-gI
sgRNA-gI-R	AAACCGGGGGACGCGGGGTCGTAC	
sgRNA-US2-F	CACCGGGGGTGACGGCCATCACCG	sgRNA-US2
sgRNA-US2-R	AAACCGGTGATGGCCGTCACCCCC	
gI/US2-F	ACCACCGCCGCGCCGGGCGTCTCGCGCCAC	Identify the deleted genes
gI/US2-R	GGCCAGCGAGCCGGGGGAGATCTCCGAGGA	

### Generation of PRV GDFS-delgI/gE/US9/US2 using CRISPR/Cas9

The homologous recombination and CRISPR/Cas9 technology were used simultaneously to gene-delete virus. Co-transfection was conducted in PK-15 cells using Lipofectamine 2000 (Invitrogen, USA) following the manufacturer’s instructions. In brief, 4 μg of pSK-gIL-US2R plasmid, 8 μg of PRV-GDFS genome, and 1 μg of pX335-sgRNAs (0.5 μg of pX335-gIsgRNA and 0.5 μg of pX335-US2sgRNA) were co-transfected to PK-15 cells as previously described. After the cytopathogenic effect (CPE), the cells were collected and subjected to 3 cycles of freezing and thawing. The PRV GDFS-delgI/gE/US9/US2-deleted virus was generated through plaque purification assay. The recombinant virus was identified by PCR test using gI/US2-F/R specific primers (Table 1), and the genetic stability was validated by consecutive culture of cells.

### Immunofluorescence assay

PK-15 cells cultured to 90% confluence in 12-well plates were infected with PRV GDFS-delgI/gE/US9/US2 at an MOI of 0.1, and the PRV GDFS wild-type strain served as the positive control. Twenty-four hours after the infection, the infected cells were fixed using cold methanol:acetone (1:1), followed by washing with PBS. Then, the cells were blocked in blocking buffer (5% bovine serum albumin in PBS) and incubated with anti-gE mAbs or anti-gB mAbs (1:100 dilution; the monoclonal antibodies were provided by Doctor Bo Hou, unpublished data. PRV gE (GenBank No. KM523549.1) or gB (GenBank No. KU552118) gene was cloned into pET28a vector. These recombinant proteins were then expressed and purified by the prokaryocyte expression system. These recombinant proteins (gE or gB protein) have been immunized in mice. Then the mouse spleen cells were fused with cell line SP2/0 cells in order to prepare monoclonal antibody. Eventually the monoclonal antibodies (anti-gE or anti-gB mAbs) were prepared). After washing three times with PBS, the cells were incubated with FITC-conjugated goat anti-mouse antibody (1:500 dilution, ABclonal, Wuhan, China). The cells were investigated under a fluorescent microscope (Olympus IX73, Japan).

### Animal experiments

#### Safety experiment

Twenty 5- to 7-day-old suckling piglets were purchased from a PRV-negative pig farm. The suckling piglets were confirmed to be seronegative for PRV using a PRV-specific gE and gB antibody ELISA kit (IDEXX, USA) and randomly divided into four groups of five. The piglets in Group A (negative-control group) were injected intramuscularly with 1 mL of DMEM. The piglets in Group B (positive-control group) were injected intramuscularly with a single dose of commercial Bartha-K61 vaccine (10^5^TCID_50_/Dose). The piglets in Group C were injected intramuscularly with 10^5^ TCID_50_ PRV GDFS-delgI/gE/US9/US2. The piglets in Group D were injected intramuscularly with 10^6^ TCID_50_ PRV GDFS-delgI/gE/US9/US2. The pigs were housed in the negative-pressure facility of Wuhan Keqian Biology Co., Ltd (Wuhan, China), and the different groups were placed in separate rooms to avoid cross infection. All the experimental materials were strictly checked to avoid cross pollution [26]. Before formal experiments, the pathogenic microorganisms such as PCV2, PRRSV, CSFV, etc. in the pigs had been checked through the real time quantitative PCR [27]. All the pigs were checked daily for their rectal temperature, and clinical signs (respiratory symptoms: sneezes, breathlessness, and nasal discharges; neurologic symptoms: opisthotonos and ataxia) were recorded throughout the experiment.

#### Efficacy experiment

Twenty 5- to 7-day-old suckling piglets free of PRV antibodies were randomly divided into four groups, with five piglets per group. The pigs in Group A (negative-control group) were injected intramuscularly with 1 mL of DMEM. The piglets in Group B (positive-control group) were injected intramuscularly with a single dose of a commercial Bartha-K61 vaccine (HIPRA, Spain). The piglets in Group C were injected intramuscularly with 10^5^ TCID_50_ PRV GDFS-delgI/gE/US9/US2. The piglets in Group D were injected intramuscularly with 10^6^ TCID_50_ PRV GDFS-delgI/gE/US9/US2. The pigs were housed in the negative-pressure facility of Wuhan Keqian Biology Co., Ltd (Wuhan, China), and the different groups were placed in separate rooms to avoid cross infection. All the experimental materials were strictly checked to avoid cross pollution [26]. Before formal experiments, the pathogenic microorganisms such as PCV2, PRRSV, CSFV, etc. in the pigs had been checked through the real time quantitative PCR [27]. All the piglets at 28 days post-primary immunization (DPI) were challenged intranasally with a 10^8.0^ TCID_50_ dose of the virulent PRV GDFS strain.

After the PRV challenge, all the piglets were checked daily for their rectal temperature, and clinical signs (respiratory symptoms: sneezes, breathlessness, and nasal discharges; neurologic symptoms: opisthotonos and ataxia) were recorded throughout the experiment. The body weights of all the pigs were individually measured at 0 days post-challenge (DPC) (challenge) and 14 DPC (necropsy). The average weight gain was calculated and analyzed.

### Serological tests

Serum samples were collected at 0, 7, 14, 21 and 28 DPI, and the PRV-specific gB and gE antibodies in the serum were detected using ELISA kits (IDEXX, USA) according to the manufacturer’s instructions.

The serum neutralization test was performed as described previously [17]. Fifty microliters of serum samples were serially diluted twofold and mixed with a 100 TCID_50_ concentration of the PRV GDFS strain or PRV Ea strain at 37 °C for 60 min. The mixture was added to confluent PK-15 cells cultured in 96-well plates and then incubated at 37 °C under 5% CO_2_ for 4 days. The cells were investigated under a microscope for the CPE. The titers of neutralization antibodies were calculated as the reciprocals of the highest serum dilutions at which no CPE was observed [17].

### Viral shedding

Nasal swab samples were collected at 0, 2, 4, 6, 8, 10, 12 and 14 DPC, and were clarified through centrifugation. The supernatant samples were passed through a sterile 0.22-micron filter and serially diluted tenfold. Then, the diluted samples were used to inoculate PK-15 cells on 96-well culture plates. The viral titers of the nasal swab samples were calculated as the TCID_50_.

### Necropsy and histopathological examination

At 14 DPC, piglets from each group were euthanized. A complete necropsy of each animal was performed. Samples were collected and fixed in 10% neutral-buffered formalin. The tonsils and brain were histopathologically examined with HE staining.

### Statistical analysis

Statistical analysis was conducted using the GraphPad prism 6.0 (GraphPad Software, USA). One-way ANOVA was used for statistical analyses among different groups. *P* < 0.05 was defined as statistically significant difference.

## Results

### Generation of the recombinant virus PRV GDFS-delgI/gE/US9/US2

The recombinant virus PRV GDFS-delgI/gE/US9/US2 was constructed by co-transfection with the PRV-GDFS genome, pX335-sgRNAs, and pSK-gIL-US2R plas-mid. At 72 h post-transfection, the cytopathic viruses were collected and purified through three rounds of plaque assays. The purified viruses were identified by PCR assays with specific primers (gI/US2-F and gI/US2-F). A specific 3964 bp fragment covering the gI/gE/US9/US2 genes was detected in the wild-type PRV GDFS strain, but the 580 bp fragment was identified in the recombinant virus, in which the gI/gE/US9/US2 genes were deleted (Fig. 1B). The 580 bp fragment of genes deleted was validated by gene sequencing. Therefore, the purified and identified genes deleted in gI/gE/US9/US2 were named PRV GDFS-delgI/gE/US9/US2.

**Fig. 2:**
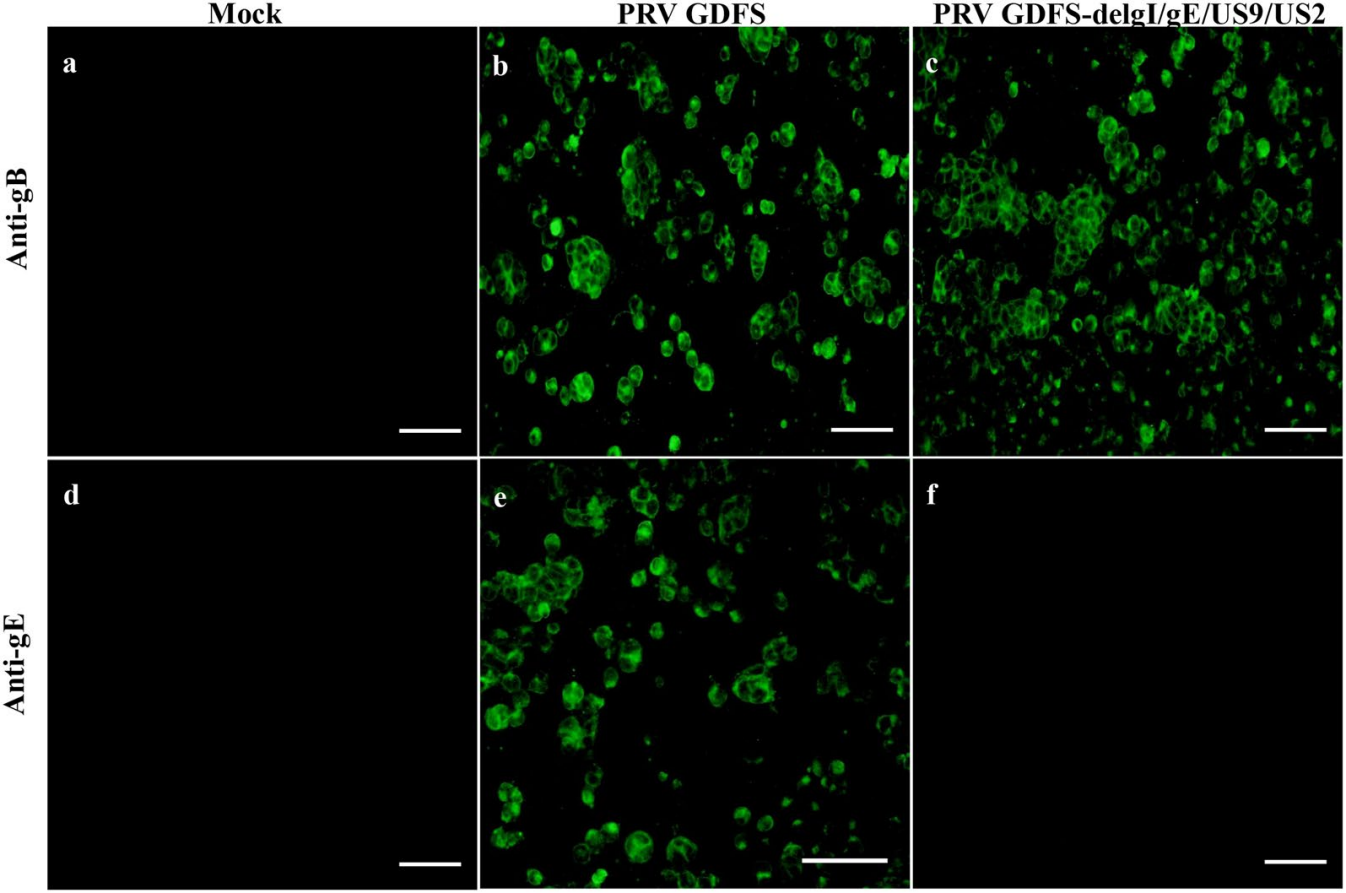
Identification of PRV GDFS-delgI/gE/US9/US2 by immunofluorescence assay. PK-15 cells were infected with either the PRV GDFS strain or the PRV GDFS-delgI/gE/US9/US2 strain and were analyzed by IFA using anti-gB MAb or anti-gE MAb as the primary antibody and FITC-conjugated goat anti-mouse antibody as the secondary antibody. Scale bar indicates 100 μm

PK-15 cells were infected with recombinant virus PRV GDFS-delgI/gE/US9/US2 strain or wild-type PRV GDFS strain to clarify the absence of the gE gene in the recombinant virus. As shown in Fig. 2, gB protein was detected by IFA in PK-15 cells infected with the PRV GDFS and PRV GDFS-delgI/gE/US9/US2, whereas the gE protein was only detected in PK-15 cells infected with PRV GDFS. These results indicated that the recombinant virus have deleted the genes of gI/gE/US9/US2.

### Safety experiment of PRV GDFS-delgI/gE/US9/US2 in suckling piglets

In investigating the safety of PRV GDFS-delgI/gE/US9/US2 as a potential live attenuated vaccine, 5- to 7-day-old suckling piglets were inoculated intramuscularly with the recombinant virus. The DMEM group is the negative control group, whereas Bartha-K61 vaccine (10^5^ TCID_50_/Dose) is the positive control group. All the suckling piglets were normal, and no clinical signs were observed throughout the experiment (Table 2). The rectal temperatures of all the suckling piglets inoculated intramuscularly with the recombinant virus were below 40.0 °C. The results indicated that doses of 10^5^ TCID_50_ or 10^6^ TCID_50_ PRV GDFS-delgI/gE/US9/US2 were safe for suckling piglets.

**Table 2:** The safety of PRV GDFS-delgI/gE/US9/US2 in 5–7 days-old suckling piglets

Groups	Doses TCID_50_/ml	Amounts	Temperature (≥ 40.0 °C)	Clinical signs
DMEM	1 ml	5	0/5	0/5
Bartha K61	10^5^	5	0/5	0/5
GDFS-delgI/gE/US9/US2	10^5^	5	0/5	0/5
	10^6^	5	0/5	0/5

### Antibody production in piglets

**Fig. 3:**
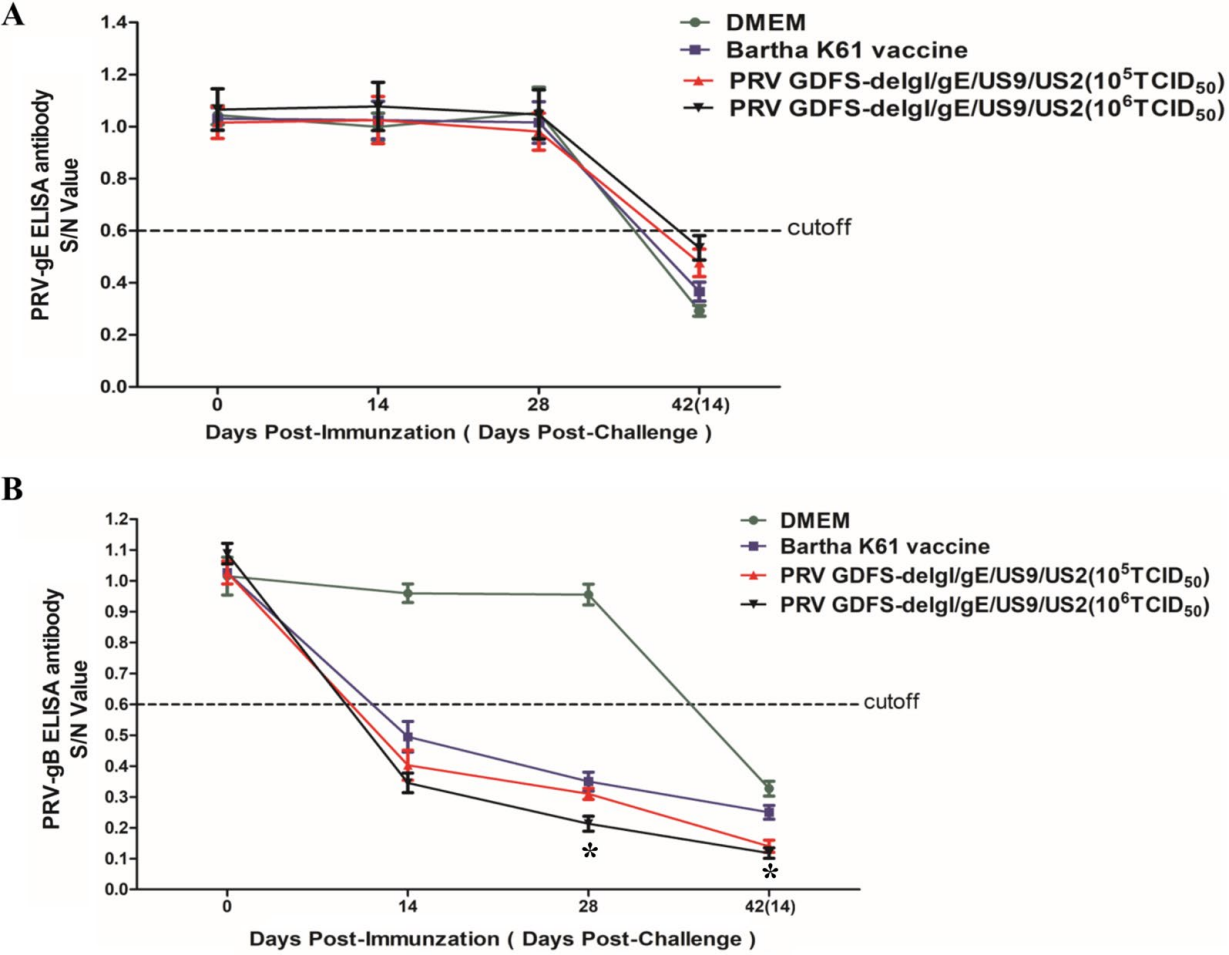
Detection of PRV-specific antibodies in immunized pigs using ELISA. Mean PRV gE-specific antibody responses (**A**) and PRV gB-specific antibody responses (**B**) in different groups. ELISA results are expressed as S/N values. Samples with S/N values less than 0.6 were considered positive. Samples with S/N values greater than 0.7 were considered negative. All data are expressed as mean ± SEM. The asterisks (*) indicate statistically significant differences (*P* < 0.05)

PRV gE-specific antibodies were measured using a competitive ELISA kit. None of the groups produced gE-specific antibodies before challenge (Fig. 3A). After the PRV GDFS wild-type strain challenge, the gE-specific antibodies were detected in all the groups at 14 DPC. However, the gE-specific antibody levels in the 10^5^ TCID_50_ or 10^6^ TCID_50_ PRV GDFS-delgI/gE/US9/US2 vaccine groups were significantly lower than those in the Bartha-K61 vaccine group.

PRV gB-specific antibodies were also measured using a competitive ELISA kit. At 14 DPI, the gB-specific antibodies in the vaccination groups were measured (Fig. 3B). The antibody levels of all the vaccinated pigs peaked at 28 DPI. No significant differences in antibody levels were detected between the 10^5^ TCID_50_ PRV GDFS-delgI/gE/US9/US2 and 10^6^ TCID_50_ PRV GDFS-delgI/gE/US9/US2 vaccine groups. However, the difference between the 10^6^ TCID_50_ PRV GDFS-delgI/gE/US9/US2 and Bartha-K61 vaccine groups was significant at 28 DPI and 14 DPC (*P* < 0.05). The gB-specific antibodies were not detectable in the DMEM group before challenge.

**Fig. 4:**
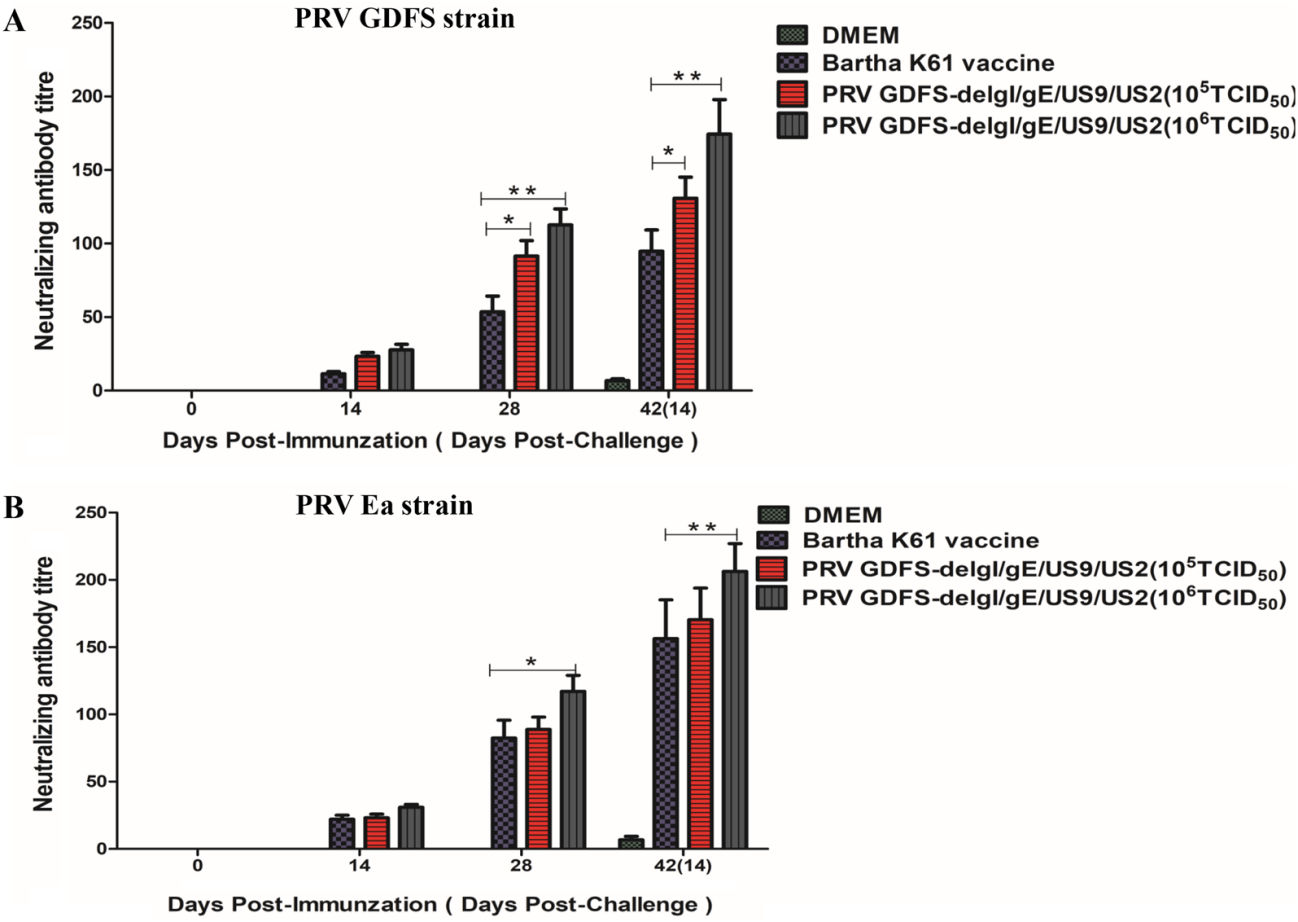
Detection of neutralizing antibodies in immunized pigs. The neutralizing antibody titers against the PRV GDFS strain (**A**) or PRV Ea strain (**B**) were calculated and are expressed as the highest dilutions that resulted in complete inhibition. All data are expressed as mean ± SEM. The asterisk (*) indicates statistically significant differences between different groups (*P* < 0.05)

The serum samples were further evaluated to determine their ability to neutralize PRV using a neutralizing test. Their neutralization activity against the two different PRV strains was detected in the DMEM group before challenge. The 10^5^ TCID_50_ or 10^6^ TCID_50_ PRV GDFS-delgI/gE/US9/US2 vaccine group showed high neutralizing titers against the PRV GDFS strain (variant PRV strain) or PRV Ea strain (older PRV strain) (Fig. 4). The neutralization titers peaked at 28 DPI in the vaccination groups. A significant difference between the PRV GDFS-delgI/gE/US9/US2 and Bartha-K61 vaccine groups in the neutralizing antibody titer was observed. At 28 DPI, the mean neutralization titers induced by the Bartha-K61 vaccine against the PRV Ea strain were higher than those against the PRV GDFS strain.

### Protection of immunized piglets against PRV challenge

**Fig. 5:**
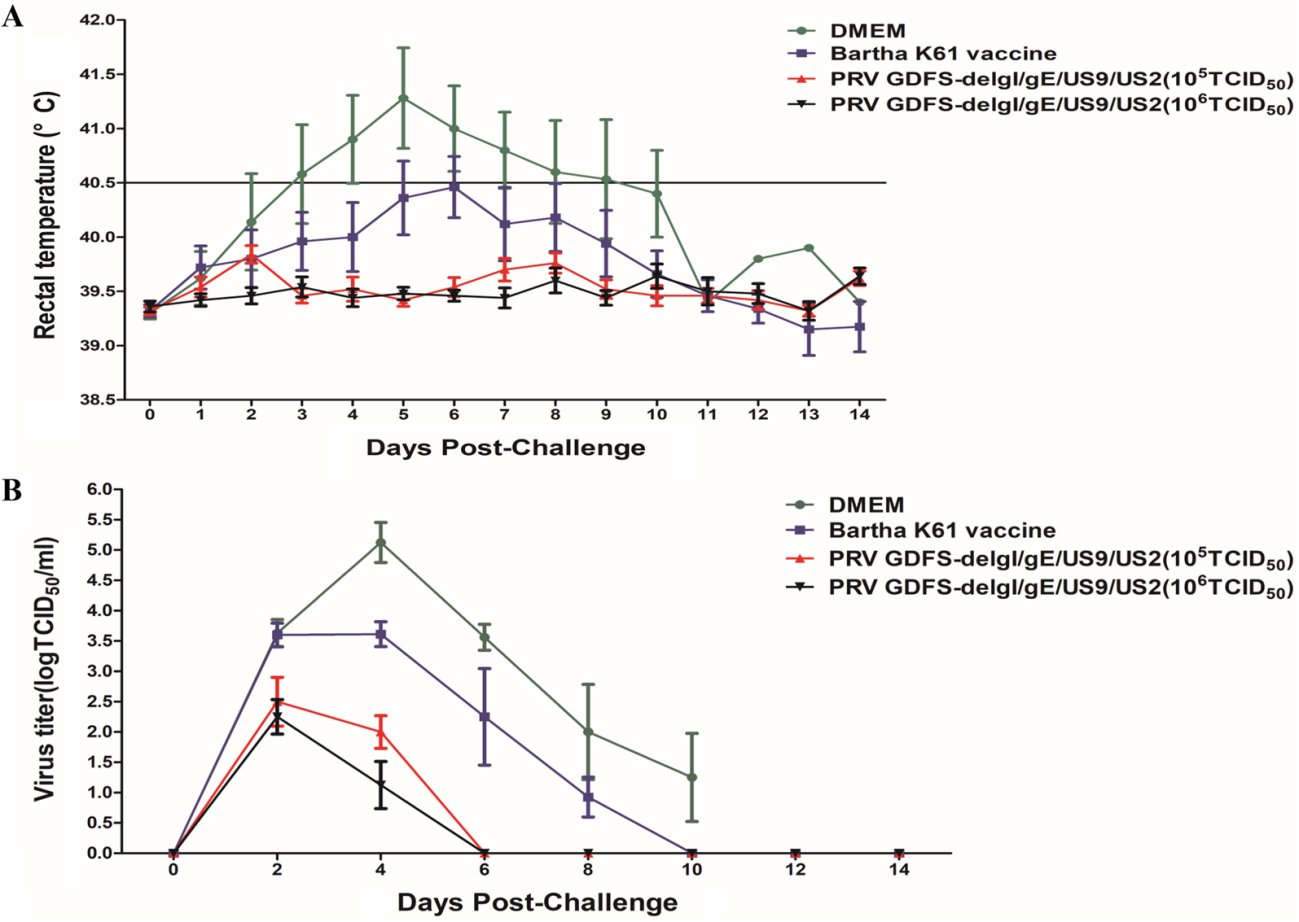
Rectal temperatures (**A**) and viral shedding (**B**) of the piglets after PRV GDFS challenge. After PRV challenge, the rectal temperature was recorded, and fever was considered as rectal temperature > 40.5 °C. Viral shedding was detected by viral isolation and expressed as TCID_50_. All data are expressed as mean ± SEM

The piglets of all the groups were intranasally challenged with 10^8.0^ TCID_50_/ml of the PRV GDFS strain at 28 DPI. The rectal temperatures of all the piglets were measured. In the DMEM group, all the pigs displayed typical clinical signs (sneezes, breathlessness, loss of appetite, and dystaxia) with high fever (> 41 °C), and all the pigs in the DMEM group died at 7–12 DPC (Fig. 5A). Three of the five pigs of the Bartha-K61 vaccine group showed fever at 3 and 7 DPC (ranging from 40.5 °C to 41.6 °C) and exhibited clinical signs, such as a loss of appetite and sneezes. However, the pigs in the Bartha-K61 vaccine group survived after the PRV GDFS strain challenge. The rectal temperatures of all the piglets immunized with 10^5^ TCID_50_ or 10^6^ TCID_50_ of the PRV GDFS-delgI/gE/US9/US2 vaccine were below 40.0 °C after PRV GDFS challenge (Fig. 5A). Moreover, no clinical signs were observed in pigs immunized with 10^5^ TCID_50_ or 10^6^ TCID_50_ of the PRV GDFS-delgI/gE/US9/US2 vaccine.

Nasal swab samples were collected after the challenge, and the viral shedding was detected by viral isolation. As shown in Fig. 5B, the titers of shed PRV were deter-mined in all the groups. The titers of the excreted PRV in the DMEM group were higher than those in the vaccinated group. The titers of the excreted PRV in the Bartha-K61 vaccine group peaked at 4 DPC, and the average PRV titer was 10^3.65^ TCID_50_/ml. The PRV shedding in the Bartha-K61 vaccine group was higher than that detected in the PRV GDFS-delgI/gE/US9/US2 vaccine groups. In addition, the difference between these 10^5^ TCID_50_ or 10^6^ TCID_50_ PRV GDFS-delgI/gE/US9/US2 vaccine groups was not significant. The piglets in the 10^5^ TCID_50_ or 10^6^ TCID_50_ PRV GDFS-delgI/gE/US9/US2 vaccine groups had already stopped shedding PRV at 6 DPC. However, PRV shedding in the Bartha-K61 vaccine group could still be detected at 8–10 DPC.

**Fig. 6:**
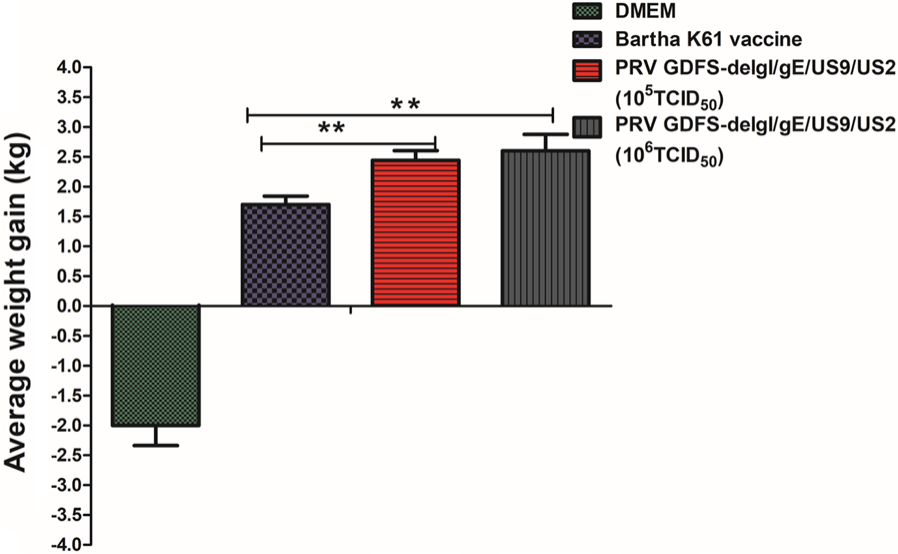
Weight gain of piglets in all groups was recorded after PRV GDFS challenge. Body weights of all pigs were individually measured at 0 DPC (challenge) and 14 DPC (necropsy). All data are ex-pressed as mean ± SEM. The asterisk (*) indicate statistically significant differences between dif-ferent groups (*P* < 0.05)

After the challenge, the piglets in the DMEM group showed poor growth and weight loss, whereas the piglets in all the vaccinated groups showed weight gain. The average weight gain in the Bartha-K61 vaccine group was significantly lower than that in the 10^5^ TCID_50_ or 10^6^ TCID_50_ PRV GDFS-delgI/gE/US9/US2 vaccine groups (Fig. 6).

### Histopathological examination

**Fig. 7:**
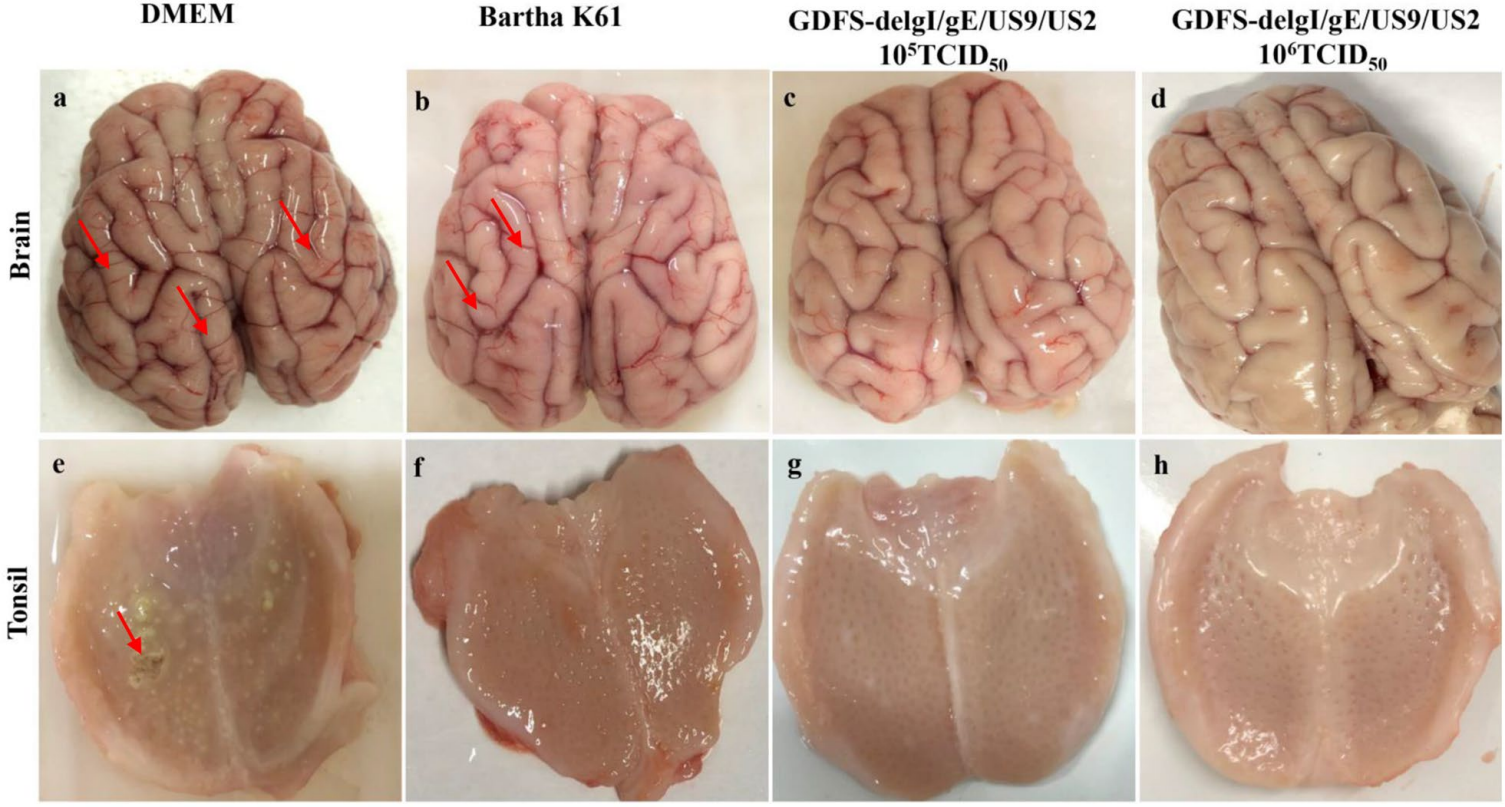
Pathological changes in piglets after PRV GDFS challenge. Autopsy was performed on all dead and surviving piglets. The brains and tonsils were collected from the pigs and subjected to pathological observation. The red arrow represents cerebral vascular hemorrhage in the brain or ulcer of the tonsil

**Fig. 8:**
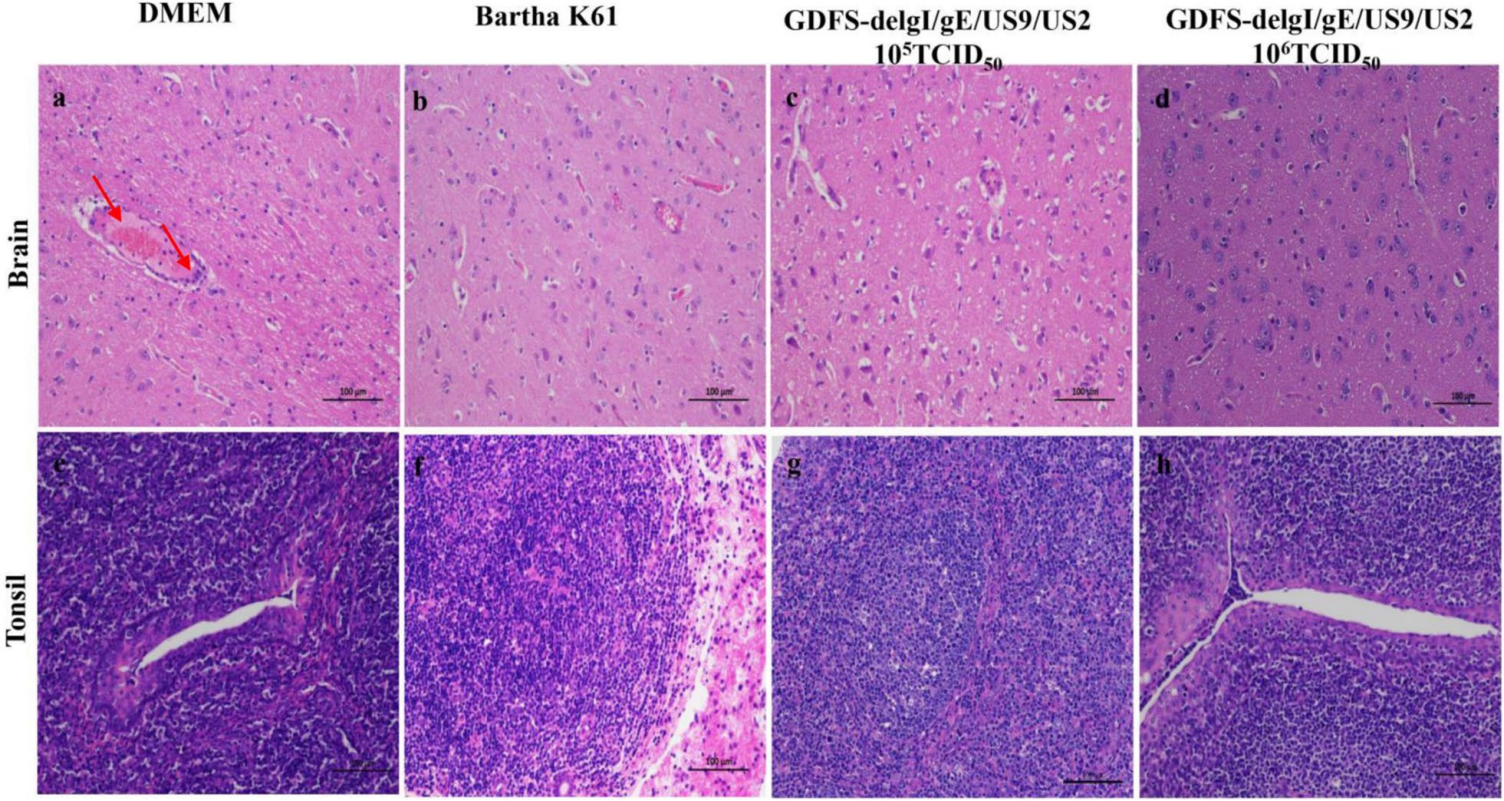
Histopathological examination of the brains (**a**–**d**) and tonsils (**e**–**h**) of piglets after PRV GDFS challenge. Original magnification is × 200. The red arrow represents perivascular lymphocyte infiltration and hemorrhage in the brain

Autopsies were performed on all the dead and surviving piglets. No apparent gross lesions were found in the 10^5^ TCID_50_ or 10^6^ TCID_50_ PRV GDFS-delgI/gE/US9/US2 vaccine groups (Fig. 7). All the dead piglets in the DMEM group showed severe brain hemorrhage and ulcers of the tonsil. No visible gross lesions in the tonsil were observed in the Bartha-K61 vaccine group, but all the piglets showed slight hemorrhages in the brain. Histopathological analyses were further performed in the brain and tonsils. The histopathological lesions in the brains of the Bartha-K61 vaccine group showed perivascular lymphocyte infiltration and hemorrhage. The piglets in the DMEM group had peri-vascular lymphocyte infiltration, hemorrhage, and necrosis in the brain. Meanwhile, a large number of inflammatory cells were found in the tonsil. By contrast, no histopathological changes were observed in the 10^5^ TCID_50_ or 10^6^ TCID_50_ PRV GDFS-delgI/gE/US9/US2 vaccine groups (Fig. 8).

## Discussion

PR is considered to be an economically important disease affecting the swine industry. Vaccines are currently an economical and effective method for controlling PRV infection in China. Since 2011, mass outbreaks of PR in China have occurred in many pig farms even after vaccination with the Bartha-K61 vaccine [13]. Various studies and clinical applications have shown that the Bartha-K61vaccine only provides partial protection to vaccinated pigs against the emerging PRV variants [3, 12, 15, 24, 28, 29]. Therefore, there is an urgent need to develop a safe and effective PRV vaccine to control the emerging PRV variants.

In our earlier research, we found that the CRISPR/Cas9 and Cre/Lox system could be used to develop a new PRV vaccine [20]. Afterward, the CRISPR/Cas9 method was widely applied to edit PRV. The gene editing system dramatically increased the efficiency of the gene-deleted PRV strain. On this basis, Yan-Dong Tang et al. (2018) reported that CRISPR/Cas9 coupled with two sgRNAs could produce a 100% knockout of PRV genes, thus providing an effective and powerful tool for PRV editing [30, 31].

In this study, we used two sgRNAs to remove nonessential genes between the two sgRNA target regions; gI, gE, Us9, and Us2 are genes not essential for PRV replication. We developed a novel gI/gE/US9/US2-deleted attenuated vaccine on the basis of the variant PRV strain (PRV GDFS), with the deletion of genes in the Bartha-K61 genome using modified CRISPR/Cas9 technology [30, 31]. Genes such as gE, gI, US9, and US2 are not essential for viral replication. The deletion of these genes results in an attenuated phenotype in vivo. A live vaccine candidate must be safe and immunogenic. If the irrational and inappropriate use of live attenuated PRV vaccines in pigs with wild-type virus circulation is not controlled, there is a risk of viral recombination [32]. The deletion in the gene encoding gE, a non-essential glycoprotein in PRV, provides a serological marker that can easily differentiate between vaccinated and infected animals. After the first round of plaque purification, these purified recombinant viruses were identified by PCR and gene sequencing. The results showed that the recombinant viruses produced a 100% knockout. The recombinant viruses were purified by three rounds of plaque assays, and its safety and efficacy were evaluated.

The safety of the vaccine was our first consideration in developing a PRV live attenuated vaccine. Suckling piglets are generally vulnerable to PRV infection, which causes high mortality and even up to a 100% death rate for infected piglets. Suckling piglets are generally preferred for the evaluation of the safety of PRV live-attenuated vaccines. In this study, 5- to-7-day-old suckling piglets inoculated with 10^5^ TCID_50_ or 10^6^ TCID_50_ PRV GDFS-delgI/gE/US9/US2 were found to be normal, and no clinical signs were observed throughout the experiment. The rectal temperatures of all the suckling piglets inoculated intramuscularly with the PRV GDFS-delgI/gE/US9/US2 vaccine were below 40.0 °C. These results indicate that PRV GDFS-delgI/gE/US9/US2 is safe for suckling piglets.

At present, conventional PRV live attenuated vaccines have the ability of differential diagnosis, which allows differentiation of vaccinated from infected animals (DIVA). Therefore, DIVA strategies are performed by PRV gE-deleted vaccines combined with PRV gE-ELISA. In our study, the piglets immunized with 10^5^ TCID_50_ or 10^6^ TCID_50_ PRV GDFS-delgI/gE/US9/US2 did not produce PRV gE-specific antibodies before challenge. This result indicated that PRV GDFS-delgI/gE/US9/US2 vaccine could serologically differentiate vaccinated animals from infected animals. On the contrary, all groups, except for the DMEM group, generated PRV gB-specific ELISA antibodies, and the PRV gB-antibody levels of the PRV GDFS-delgI/gE/US9/US2 vaccine group were higher than those of the Bartha-K61 vaccine group. Furthermore, the 10^5^ TCID_50_ or 10^6^ TCID_50_ PRV GDFS-delgI/gE/US9/US2 vaccine groups induced high neutralizing titers against PRV GDFS strain (variant PRV strain) or PRV Ea strain (older PRV strain). A strong association could be observed between the levels of neutralizing antibodies and protection against PRV challenge. The PRV GDFS-delgI/gE/US9/US2 vaccine showed enhanced cross-reactive neutralization antibodies against variant PRV strain or older PRV strain.

In accordance with the manual of diagnostic tests and vaccines for terrestrial animals, the efficacy of PRV vaccines was evaluated according to the four criteria after PRV challenge [33]. Such criteria include rectal temperature, weight loss, clinical signs, and mortality. In general, a high titer of the PRV virulent strain (≥ 10^7.5^ TCID_50_/ml) is recommended. In our study, a high-dose challenge with 10^8^ TCID_50_/ml of the PRV GDFS virulent strain was performed via the intranasal route in all the groups. After the PRV challenge, none of the piglets immunized with the PRV GDFS-delgI/gE/US9/US2 vaccine showed any clinical signs and their rectal temperatures were normal. In addition, three of the five pigs in the Bartha-K61 vaccine group showed fever at 3 and 7 DPC and exhibited clinical signs such as a loss of appetite or sneezing. The piglets in all the vaccinated groups showed weight gain and no mortality. However, the average weight gain in the Bartha-K61 vaccine group was significantly lower than that in the PRV GDFS-delgI/gE/US9/US2 vaccine groups. Furthermore, the autopsy and histopathological analyses revealed that the piglets in the PRV GDFS-delgI/gE/US9/US2 vaccine groups did not show apparent gross or pathological lesions. All the piglets in the Bartha-K61 vaccine group showed slight hemorrhages and pathological lesions in the brain. After challenge with the PRV GDFS variant strain, virus shedding was detected in all the groups. The PRV shedding in the Bartha-K61 vaccine group was higher than that detected in the PRV GDFS-delgI/gE/US9/US2 vaccine groups, and the excretion of virus in the Bartha-K61 vaccine group lasted longer. The results for the virus shedding were consistent with those of previous reports stating that PRV vaccines cannot completely prevent PRV infection [21]. According to the criteria of the OIE terrestrial manual, the results of the experiment confirmed that the PRV GDFS-delgI/gE/US9/US2 vaccine could provide full protection against the emerging PRV variant strain in piglets, in contrast to the commercial Bartha-K61 vaccine.

PRV-GDFS (GenBank No. MH521043), belonging to the variant PRV strain, was isolated from the Guangdong Province of China in 2019. Homologous analysis showed that the gB or gD genes of PRV GDFS and the isolated variant strains (GII) reached 99 ~ 100%, which is consistent with the deduced amino acid sequences. Therefore, we hypothesized that the PRV GDFS-delgI/gE/US9/US2 vaccine is protective against other variant strains (GII).

## Conclusion

A novel quadruple gene-deleted vaccine based on an emerging PRV variant, which showed the deletion of the gI, gE, US9, and US2 genes, was generated using the CRISPR/Cas9 method. A safety experiment confirmed that PRV GDFS-delgI/gE/US9/US2 is safe for suckling piglets. The experiment on piglets challenged with the emerging PRV variant strain showed that the PRV GDFS-delgI/gE/US9/US2 vaccine confers complete protective immunity. In future studies, we will evaluate the efficacy and safety of the PRV GDFS-delgI/gE/US9/US2 vaccine in pregnant sows.

## Data Availability

The datasets used and analyzed during the current study are available from the corresponding author on reasonable request.

## Abbreviations

PR: Pseudorabies
PRV: Pseudorabies virus
CRISPR: Clustered regularly interspaced short palindromic repeats
DMEM: Dulbecco’s modified essential medium
PCR: Polymerase chain reaction
TCID_50_: 50% Tissue culture infective dose
DPI: Days post-primary immunization
DPC: Days post-challenge
DIV1: Differentiation of vaccinated from infected animals
OIE: World Organization for Animal Health
